# Stone disease in pregnancy: imaging-guided therapy

**DOI:** 10.1007/s13244-014-0352-2

**Published:** 2014-09-24

**Authors:** Gabriele Masselli, Martina Derme, Maria Giulia Bernieri, Elisabetta Polettini, Emanuele Casciani, Riccardo Monti, Francesca Laghi, Marialuisa Framarino-dei-Malatesta, Marianna Guida, Roberto Brunelli, Gianfranco Gualdi

**Affiliations:** 1 Department Radiology, Università di Roma Sapienza, Viale del Policlinico 155, Rome, 00161 Italy; 2 Department of Gynecology, Università di Roma Sapienza, Viale del Policlinico 155, Rome, 00161 Italy

**Keywords:** Nephrolithiasis, Pregnancy, Magnetic resonance, Ultrasound-CT therapy

## Abstract

Renal colic is the most frequent nonobstetric cause for abdominal pain and subsequent hospitalization during pregnancy. The physio-anatomical changes in the urinary tract and the presence of the fetus may complicate the clinical presentation and management of nephrolithiasis. Ultrasound (US) is the primary radiological investigation of choice. Magnetic resonance urography (MRU) and low-dose computed tomography (CT) have to be considered as a second- and third-line test, respectively. If a study that uses ionizing radiation has to be performed, the radiation dose to the fetus should be as low as possible. The initial management of symptomatic ureteric stones is conservative during pregnancy. Intervention will be necessary in patients who do not respond to conservative measures. Therefore, it is crucial to obtain a prompt and accurate diagnosis to optimize the management of these patients.

*Teaching Points*

• *In pregnancy, renal colic is the most frequent nonobstetric cause for abdominal pain and hospitalization.*

• *Magnetic resonance urography should be considered when ultrasound is nondiagnostic.*

• *Low-dose CT should be considered as a last-line test during pregnancy.*

## Introduction

Renal colic is the most frequent nonobstetric cause for abdominal pain and subsequent hospitalization during pregnancy [1, 2]. Symptomatic nephrolithiasis complicates 1 in 3,300 pregnancies with an incidence ranging from 1:200 to 1:1,500 [3, 4]. The incidence of the symptomatic cases is similar between pregnant and nonpregnant women.

The physio-anatomical changes in the urinary tract and the presence of the fetus may complicate the clinical presentation and subsequent management of nephrolithiasis [5].

Renal colic has been associated with several pregnancy complications, including preterm labor and delivery, recurrent abortions, hypertensive disorders, gestational diabetes and cesarean deliveries. These potential complications make accurate diagnosis crucial [6, 7].

In a series of pregnant patients with renal colic, 28 % of patients with abdominal pain had an incorrect admitting diagnosis based on clinical evaluation. These diagnoses included appendicitis, diverticulitis and placental abruption [8].

Ultrasound (US) is widely used as the first-line diagnostic test in pregnant women with abdominal pain because of the availability, low cost and lack of ionizing radiation.

An increasing number of studies have shown that MRI is valuable in evaluating specific causes of abdominal and pelvic pain in pregnancy and is the preferred investigation when ultrasound is inconclusive owing to the lack of ionizing radiation. [9]

Magnetic resonance urography (MRU) without contrast should be considered as a second-line test during pregnancy when use of US fails to establish a diagnosis and there are continued symptoms despite conservative management [10–13].

Low-dose computed tomography (CT) is a highly sensitive and specific diagnostic modality for detecting stones in the urinary tract, but because of the radiation exposure it should be considered as a last-line test during pregnancy [14–16].

The role of the radiologist is to provide a prompt and accurate diagnosis, to avoid late and inadequate treatment. The aim of this review is to explain the use of the different imaging techniques for the diagnosis and management of nephrolithiasis during pregnancy based on a literature review and the authors’ experience.

### Clinical presentation

Flank or abdominal pain is the most common symptom; it occurs in 85 % to 100 % of patients [17–19]. Frank hematuria is reported in 15 % to 30 % of proven cases, and microscopic hematuria can be detected in 95 % to 100 % of cases, although urinalysis may need to be repeated up to three times [20, 21].

Nonspecific symptoms, including nausea and vomiting, are sometimes present. Other modes of presentation include urinary tract infection (UTI), preeclampsia, and premature onset or arrest of labor [22, 19].

### Diagnosis

Given the established risks to the fetus from radiation exposure, ultrasound and magnetic resonance imaging (MRI) are the strongly preferred imaging investigations [23].

If ionizing radiation is used, one must adhere to the principle of using a dose that is as low as reasonably achievable after a discussion of risks versus benefits with the patient.

#### Ultrasound

US is the first investigation for all pregnant women when there is a suspicion of stone disease.

Real-time US demonstrates the renal parenchyma, pelvicaliceal system, dilated ureter and occasionally the offending calculus without fetal radiation exposure.

Doppler US can produce high intensities and should be used judiciously, keeping the exposure time and acoustic output to the lowest level possible, especially during the first trimester [24]. A temperature elevation higher than 1.5 °C is considered hazardous and can be reached during Doppler studies [25].

US is operator dependent, and sensitivity to detect nephrolithiasis during pregnancy ranges from 34 to 92.5 % [26]. Moreover, it is highly nonspecific and may be unable to differentiate between ureteral obstruction secondary to calculi and physiological hydronephrosis because of the mechanical compression of the ureter between the gravid uterus and the iliopsoas muscle.

Transvaginal US can be helpful to evaluate the distal ureter and distinguish obstruction from physiological hydronephrosis of pregnancy [27], as demonstrated by Abdel-Kader et al. in their study including 23 pregnant women with symptoms suggestive of ureteral calculi [28].

Doppler-assisted measurement of the resistive index (RI) (peak systolic velocity of intrarenal blood flow minus the end-diastolic velocity divided by the peak systolic velocity) has shown some promise in pregnancy. The RI does not appear to be affected by the physiologic hydronephrosis during pregnancy [29]. Normal pregnancy does not usually affect the intrarenal RI, and an elevated RI (>0.70) should not therefore be attributed to pregnancy [30].

Absence of a “ureteral jet” (passage of urine at the uretero-vescical junction) on the suspected side of an obstruction has been reported to have a sensitivity of 100 % and specificity of 91 % [31]. Patients should be imaged in the contralateral decubitus position to decrease false-positive results [32].

#### Computed tomography

CT, involving radiation use, should not be performed during pregnancy because of teratogenic risks and risk of childhood malignancy [33].

The use of low-dose CT for detection of calculi has been validated in the general population [34, 35]. Although low-dose multidetector CT presents a high accuracy (95 % sensitivity and 98 % specificity) for detection of calculi in the general population, it is still used as a last-line test.

A recent review by Goldberg-Stein et al. reported that the fetal dose from a single-acquisition abdominopelvic CT study had to range between 10 and 50 mGy [36]. In a previous review, the same author asserted that the risk of childhood cancer was negligible at doses of less than 50 mGy [37]. However, although the risks of teratogenesis are minimal, fetal exposure from pelvic CT within the range of 20 to 50 mGy increases the risk of fatal childhood cancer by a factor of 1.4 to 2 [38]. Therefore, high-dose ionizing radiation examinations such as CT can only be justified in pregnant women when the study is overwhelmingly in the best health interest of the mother, i.e., there is no diagnostic alternative [39].

#### Magnetic resonance urography

MRU without contrast is safe and effective, has comparable accuracy to CT and is now considered the second-line investigation during pregnancy when available. MRU can rapidly acquire images of the upper urinary tract without the administration of intravenous contrast agents [10–13].

**Fig. 1:**
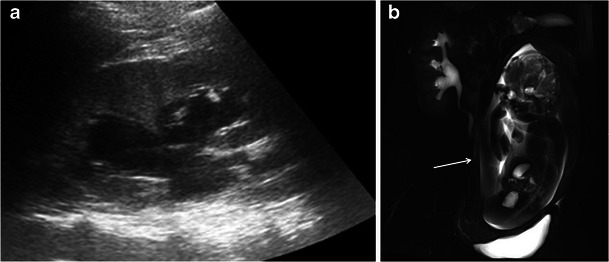
A 28-year-old woman was admitted at 38 weeks of gestation presenting with acute abdominal pain. US showed dilatation of the right pelvicaliceal system, with no visualization of the ureter (**a**). Coronal MR urography shows smooth tapering of the middle third of the ureter because of the mass effect between the uterus and the adjacent retroperitoneal musculature. This finding is characteristic of physiological urinary tract dilatation (**b**)

Magnetic resonance urography (MRU) using heavily T2-weighted ‘water’ images with thick slabs is useful to detect the urinary system and the ureters, differentiating physiological urinary tract dilatation from abnormal dilatation related to urolithiasis (Fig. 1).

Thin-slice, high-resolution, highly T2-weighted fast spin echo (FSE) sequences can improve the ability of MRU for detection of small stones [23].

In a series of 24 pregnant patients with symptomatic hydronephrosis, MRU was noted to show different appearances in physiologic hydronephrosis and pathologic obstruction [40]. Renal enlargement and perirenal fluid suggestive of obstruction were absent in physiologic dilatation. In addition, physiologic dilatation demonstrated a characteristic tapering due to extrinsic obstruction of the middle third of the ureter by the uterus [40]. Although MRI is not accurate in detecting ureteral calculi, some features may help the visualization of obstructing calculi: stones appear as signal voids overlying the high signal of urine within a dilated ureter [41]. The presence of a standing column of urine below the level of the pelvic brim, in addition to proximal ureteral dilation, suggests an obstructing distal ureteral calculus (“double kink sign”) [41]. Other MRI features that are indicative of pathologic rather than physiologic hydronephrosis include an “unusual” site of obstruction (such as the pelvic ureteral junction or vesicoureteric junction), an abrupt ending of the ureter (rather than a smooth taper at the level of the pelvic brim), and perinephric or periureteral edema [23]. In contrast, physiologic hydronephrosis is characterized by gradual, smooth tapering of the mid to distal ureter due to extrinsic compression between the gravid uterus and iliopsoas muscle.

**Fig. 2:**
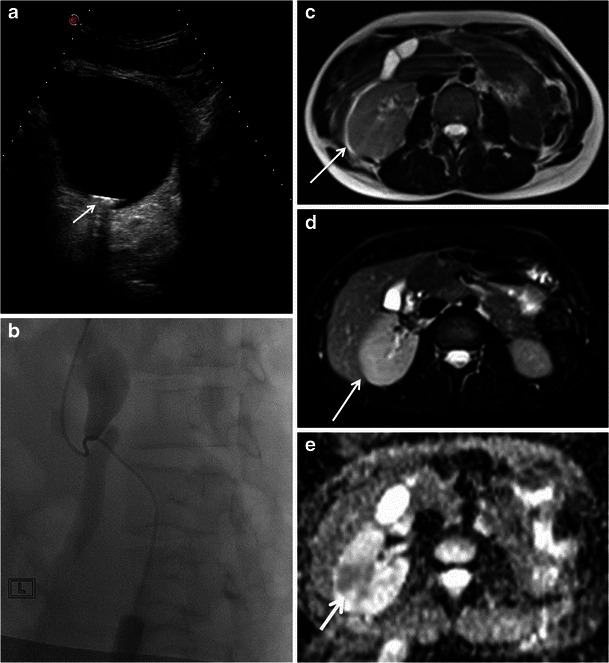
A 28-year-old woman was admitted at 35 weeks of gestation presenting renal colic not responding to medical treatment. US images showed left hydronephrosis due to a 1-cm stone impacted at the level of the ureterovesical junction (**a**). A percutaneous nephrostomy was performed and the stone extracted (**b**). Two days after this procedure, the patient presented with fever and acute pain localized to the left flank. Ultrasound was unremarkable. The axial T2-weighted HASTE sequence (**c**) and axial T2-weighted fat-saturated sequence (**d**) show an enlarged edematous right kidney; focal areas of higher signal intensity on T2-weighted imaging could be due to focal infection. ADC map (**e**) shows restricted proton diffusion indicative of pyelonephritis

MRI can also demonstrate complications such as pyelonephritis that are visualized as an enlarged edematous kidney. Areas of focal pyelonephritis show lower signal intensity on T2-weighted images and restricted proton diffusion on the DWI [42] (Fig. 2).

Limitations of MRU include limited visualization of small calculi, relatively high costs and being time-consuming. Other potential limitations of MRU include that spatial misregistration may occur between slices if the patient is breathing freely and also that T2-weighted images may develop flow-void artifacts in urine within a dilated collecting system that mimic filling defects. These flow artifacts are typically centrally located and do not layer dependently, as would be expected in a stone.

There is no scientific evidence of risk to the human fetus from MR imaging during pregnancy. MR imaging at 1.5 T or lower magnetic field strength has been used to evaluate diseases in pregnancy for over 20 years without any documented harmful effects. Therefore, the statement issued in 1991 by the Safety Committee of the Society of Magnetic Resonance Imaging that “MR imaging may be used in pregnant patients…if the examination provides important information that would otherwise require exposure to ionising radiation” is still valid today. The American College of Radiology (ACR) stated that MRI is a useful problem-solving tool in the evaluation of pelvic pain in pregnant women and, when available, MR is preferred to CT because it does not employ ionizing radiation. Pregnant women should be informed that, to date, there has been no evidence that the use of clinical MR imaging during pregnancy has produced deleterious effects. However, because of active organogenesis in the first trimester, the absolute safety of MR imaging during this period is difficult to establish. MR imaging is best avoided unless the potential benefits outweigh the theoretical risks. This statement refers to machines in clinical use at 1.5 T or less [39].

The safety of MR at 3 T has not been proven yet. However, to the best of our knowledge, there are no studies in literature documenting adverse effects on children exposed in utero to 3-T field strength.

### Treatment

The spontaneous passage rate of stones during pregnancy is up to 64–84 % of cases with conservative therapy [43–45].

A conservative approach should be the initial management in all pregnant patients with symptomatic ureteric stones. Conservative treatment, which requires close communication between the urologist and obstetrician, includes hydration (oral or intravenous), analgesia, antibiotics (if infection is present), antiemetics, rest and routine sieving of urine.

If the conservative approach fails, stenting (ureteral Double-J stent) may be performed as an initial procedure in patients who have fever and/or proximal ureteric stones. Percutaneous nephrostomy (PCN) should be reserved for patients with urosepsis or pyonephrosis if a stent cannot be placed.

Ureteroscopy with a Holmium laser has become the procedure of choice in pregnancy for symptomatic stones less than 1 cm and in those without evidence of sepsis or a history of transplanted kidney [46].

**Fig. 3:**
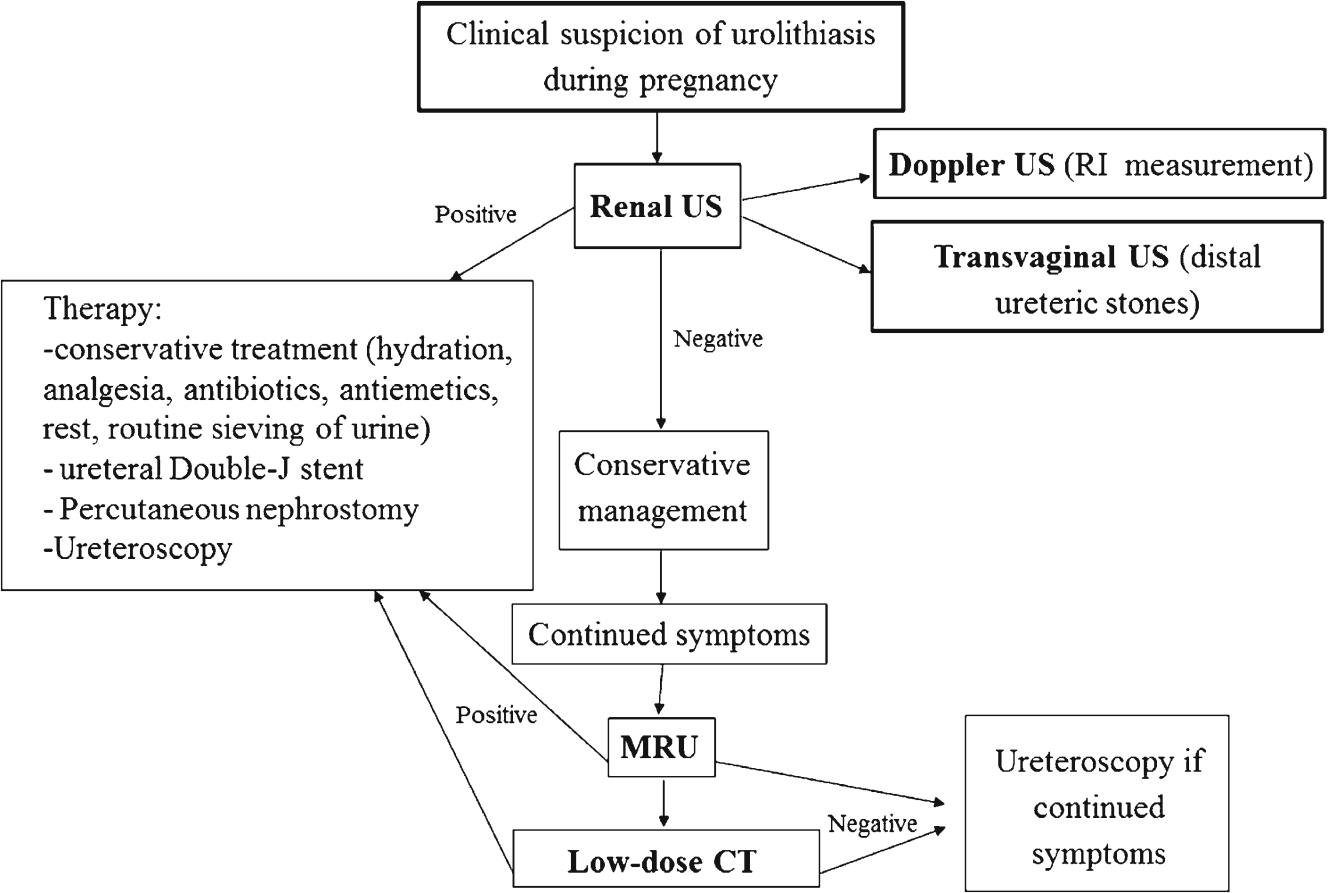
Algorithm for the management of urolithiasis during pregnancy

The algorithm for management of urolithiasis during pregnancy is summarized in Fig. 3.

## Conclusion

Urolithiasis during pregnancy is more complex than when it occurs in nonpregnant patients, and diagnosis can sometimes be quite challenging.

US is the primary radiologic investigation of choice; MRU and low-dose CT have to be considered as a second- and third-line test, respectively. If a study that uses ionizing radiation has to be performed, the radiologist has to keep the radiation dose to the fetus as low as possible [preferably below 50 mGy (i.e., 5 rad)].
